# Evolution of deterrence with costly reputation information

**DOI:** 10.1371/journal.pone.0253344

**Published:** 2021-06-15

**Authors:** Ulrich Berger, Hannelore De Silva

**Affiliations:** 1 Department of Economics, Vienna University of Economics and Business, Vienna, Austria; 2 Institute for Finance, Banking and Insurance and Research Institute for Cryptoeconomics, Vienna University of Economics and Business, Vienna, Austria; University of Electronic Science and Technology of China, CHINA

## Abstract

Deterrence, a defender’s avoidance of a challenger’s attack based on the threat of retaliation, is a basic ingredient of social cooperation in several animal species and is ubiquitous in human societies. Deterrence theory has recognized that deterrence can only be based on credible threats, but retaliating being costly for the defender rules this out in one-shot interactions. If interactions are repeated and observable, reputation building has been suggested as a way to sustain credibility and enable the evolution of deterrence. But this explanation ignores both the source and the costs of obtaining information on reputation. Even for small information costs successful deterrence is never evolutionarily stable. Here we use game-theoretic modelling and agent-based simulations to resolve this puzzle and to clarify under which conditions deterrence can nevertheless evolve and when it is bound to fail. Paradoxically, rich information on defenders’ past actions leads to a breakdown of deterrence, while with only minimal information deterrence can be highly successful. We argue that reputation-based deterrence sheds light on phenomena such as costly punishment and fairness, and might serve as a possible explanation for the evolution of informal property rights.

## Introduction

Human societies rely to a large extent on cooperation, a form of altruistic interaction where individuals pay a cost for others to receive an even higher benefit [1, 2]. While this phenomenon is readily observable, another type of cooperation is omnipresent in modern societies but hidden from direct observation: the absence of committing harmful acts towards others for one’s own benefit. Human life is full of opportunities to gain advantages by exploiting others. But for most of us the rule is that we do not seize these opportunities. Usually we do not lie, we do not steal, and we refrain from killing others for our own gain. Arguably, the absence of these win-small-lose-big interactions is as fundamental to the proliferation of civilization as the presence of lose-small-win-big interactions.

Modern societies have established a multitude of institutions such as laws, police forces, and prisons with the main purpose of providing incentives against committing hostile acts against others. In ancient human groups where formal institutions had not yet been established, informal norms of behavior served as disciplining devices, threatening deviators with punishment by group members or with ostracism [3–6]. Such a behavior is called *deterrence*: harmful acts towards others are deterred by the threat of retaliation. This mechanism is fundamental for some animal species and specifically for human societies [7–11]. It has been suggested that humans have evolved a cognitive “revenge system” that implements this deterrence strategy [12].

**Fig 1:**
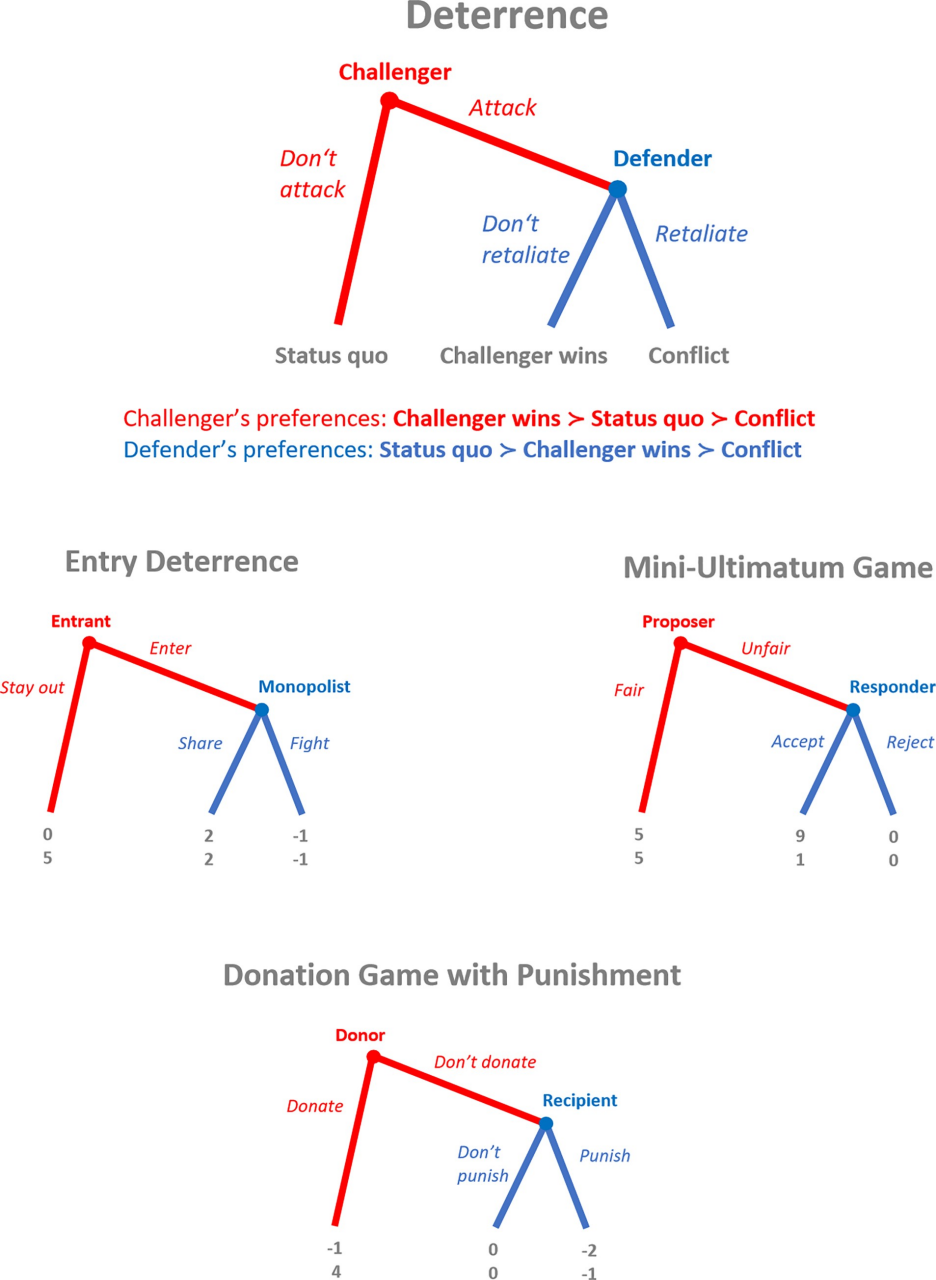
Three special cases of deterrence. In the Entry Deterrence Game (EDG) of economics textbook fame a potential entrant into a monopolized market can stay out or enter the market. If he enters, the (former) monopolist can share the market or start a price war that is detrimental to both competitors’ profits. In the Mini-Ultimatum Game the proposer has to divide a sum of 10 dollars between himself and the responder. A fair division is always implemented. An unfair division may be accepted or rejected by the responder. In the latter case, no one gets anything. In the Donation Game with Punishment, the donor has to decide whether or not to donate to a needy recipient. If he doesn’t donate, the recipient may (costly) punish the donor. In all three cases, the retaliation threat is not credible in a one-shot interaction.

The most basic form of deterrence happens in a one-to-one interaction between a challenger and a defender, to use the terminology of classical deterrence theory. The challenger decides whether or not to attack the defender. If he refrains from attacking, the status quo is upheld. If he attacks, the defender decides whether or not to retaliate. If she does not retaliate, the challenger wins. If she retaliates, conflict ensues. For both parties conflict is the worst outcome, but the challenger prefers himself winning to the status quo, while the defender would rather maintain the status quo than let the challenger win. The incentive structure of the deterrence game is general enough to comprise several well known games as special cases. Specifically, these include the Entry Deterrence Game, the Mini-Ultimatum Game and the Donation Game with Punishment, see Fig 1. Deterrence is successful if the challenger expects the defender to retaliate if attacked and therefore refrains from attacking. This simple logic, however, suffers from the lack of credibility of the threat of retaliation. While retaliation is costly to the attacker, it is also costly to the defender. In a one-shot interaction, a defender who has been harmed by a challenger’s attack thus gains nothing by retaliating and the threat of retaliation remains empty [13–15]. This raises the question how successful deterrence might have evolved at all.

If defenders interact repeatedly with different challengers and interactions are observed by others, there is an opportunity for defenders to establish a reputation of being retaliatory by carrying out the threat even though doing so is costly in the short run. If challengers can obtain information on defenders’ past behavior and tend to avoid attacking “tough” opponents, this strategy might pay off in the long run [16–18]. For the Mini-Ultimatum Game- and the Donation Game with Punishment-incarnations of the Deterrence Game (see Fig 1) reputation-based deterrence has indeed been established in various evolutionary models, e.g. [19–24]. But in these models, information on past behavior of defenders is exogenously provided to (some) members of the population at no cost. Moreover, defenders are restricted to use only deterministic strategies, either always retaliating or never. Under these assumptions, a challenger who is informed of some past action of his current opponent can perfectly anticipate her reaction to an attack. This effectively reverses the sequence of decisions. For informed challengers, the conditional strategy of not attacking retaliators becomes optimal, providing a straightforward solution to the deterrence paradox. But assuming that information on reputation just falls from heaven has been criticized as”hard to justify” [25].

Here we assume that information on defenders’ reputation has to be actively and costly acquired by challengers. Whether or not this information is collected is a strategic decision that is endogenously determined within the model. This is not only the more realistic case, but also the more difficult one for establishing deterrence. For example, if a defender population tends to homogenize, with most defenders behaving the same way, then it ceases to pay for challengers to invest in knowledge about their individual opponent’s past behavior. This again destroys defenders’ incentives to uphold a tough reputation and thereby diminishes the prospects of successful deterrence.

We show analytically that the fate of a population interacting in the deterrence game depends strongly on the economic microfoundations of reputation. If challengers are only informed of the last action of their defender, deterrence can often evolve. But if they can obtain precise information on the defender’s (possibly stochastic) strategy, deterrence inevitably breaks down. Agent-based simulations further corroborate that the prospects for deterrence decline with the amount of information provided to challengers.

## Results

To study the mechanism of deterrence and its possible evolution, we first develop a simplified dynamic game-theoretic model. Consider a large population of individuals who are repeatedly randomly matched in pairs. In each matched pair, one individual is randomly chosen to play the role of the challenger and the other one to play the role of the defender. For concreteness, we frame the situation as one involving the owner of a valuable resource like food or a tool in the defender’s role and a random passerby who has the opportunity to take away this resource in the challenger’s role. The challenger may either take (*T*) the defender’s resource or respect (*R*) her property. If he respects, his payoff is 0, while the defender keeps her resource of value *v*.

**Fig 2:**
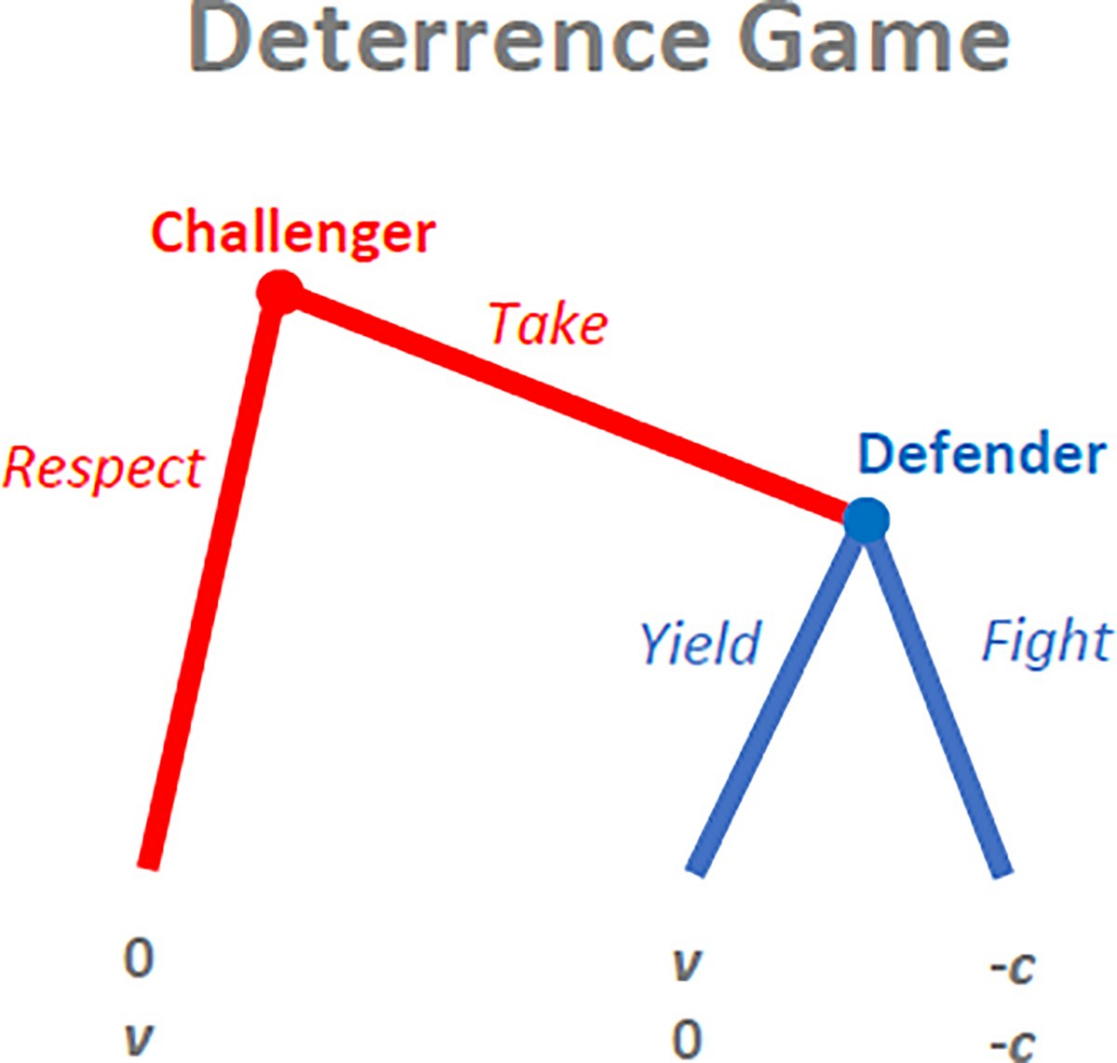
The ownership variant of the deterrence game. If the challenger respects the defender’s property of value *v*, the defender keeps it and the game ends. If the challenger takes possession of the defender’s property and she yields, her property changes hands. If the defender fights, both parties incur an expected net loss of *c*.

If the challenger takes, the defender has two possible ways to react. If she yields (*Y*), her resource changes hands and the payoffs are *v* for the challenger and 0 for herself. If she fights back (*F*), each individual ends up with the resource with probability 1/2 in the resulting conflict, but the costs are larger than this expected gain, such that the net expected payoff of a fight is −*c* for both opponents. Fig 2 shows the sequence of actions and the payoffs in this version of the deterrence game. Note that this game seems less general than the ordinal deterrence game from Fig 1, since to keep the number of parameters to a minimum the value of the resource and the costs of conflict are assumed to be the same for both parties. However, our analytical treatment indeed applies to the general payoff structure of the deterrence game (see S1 Appendix).

In a one-shot interaction, yielding is the optimal reaction to taking, and it is also optimal to take from a yielder. (*T*,*Y*) is therefore a Nash equilibrium where deterrence fails. Successful deterrence, on the other hand, requires the strategy combination (*R*,*F*). This is also an equilibrium, since respecting is optimal when meeting a defender who is predisposed to fight, and at the same time respecting renders both the defender’s (planned) reactions outcome-equivalent and therefore optimal for the challenger. However, this deterrence equilibrium is based on the defender’s empty threat to fight if her resource is taken and is thus unlikely to materialize; in game theoretic terms it lacks subgame perfection. The same also holds for the range of mixed equilibria where the challenger respects and the defender plans to fight with a probability high enough to render respecting optimal.

### Costly observation of past behavior

Without any supporting mechanism, deterrence cannot evolve. As long as some takers are present, yielders will always gain more than fighters, and once fighters have largely disappeared, takers take over the population. Observation of a defender’s reputation in the form of information about her past behavior opens up the possibility of using a discriminatory strategy by taking from “weak” defenders and respecting “tough” ones. This suggests a mechanism allowing deterrence to evolve. We therefore assume that a challenger meeting a defender can opt to obtain information on the defender’s reputation prior to deciding whether or not to take. Reputations are binary and defenders can be classified as *tough* or *weak*. In previous reputation models [19–24], information was assumed to be public or to be observed with a fixed exogenous probability at no cost. In our model, observing a defender’s reputation is a challenger’s *choice*, and it is costly to obtain this information. We assume that challengers who check a defender’s reputation incur a cost *a*. In medium-sized real populations this cost is likely to be small, as taking part in gossip is often enough to obtain the required information.

We consider three different strategies for challengers. The first two are not to check the defender’s reputation and just always to respect (*AllR*) or always to take (*AllT*). The third is the discriminator strategy that checks the defender’s reputation and then takes if and only if the defender is weak (*Disc*). The three remaining possible modes of reacting to obtained reputation information are indiscriminate respecting (*IR*), indiscriminate taking (*IT*), and the paradoxically anti-discriminating strategy of taking only from tough defenders (*Par*). These are strictly dominated, however, and will not survive in a population for long. We therefore disregard these strategies in our analytical model, but we include them for agent-based simulations.

Instead of always yielding or always fighting, defenders may also play a *randomized* stationary strategy, in each interaction fighting with some specific probability. This is important because randomizing might be a way to balance the need for fighting to obtain a tough reputation and the need for yielding to avoid the costs of conflict. We therefore assume that a defender can choose her fighting probability *Q* from the interval [0,1] which includes the pure strategies of always fighting and always yielding. We call such an individual a *Q*-defender. Of special importance is the fighting probability $Q^{*}=\frac{v}{c+v}$ that leaves challengers just indifferent between taking and respecting

### Reputation assessment schemes

Assessment of a defender’s reputation is undisputed for non-randomizing defenders: pure fighters will have a tough reputation and pure yielders a weak one. But there are many different ways to classify defenders who randomize. We consider two different simple schemes that map past behavior to reputations. Under the first one, called *last-action*, a discriminator observes the defender’s last action and classifies her accordingly. This scheme is a simple analog to the (binary) image scoring scheme of indirect reciprocity [26, 27], but it differs from that one insofar as it includes rounds with inaction by the defender, which is not applicable in the image scoring scheme. In our model, you get a tough reputation if you fight and a weak reputation if you yield, while you keep your previous reputation if your property is respected.

An important implication is that a defender’s reputation depends not only on her fighting probability *Q* but also on the state of the challenger population. If there are many takers, a defender has to react frequently and in any round her probability of having a tough reputation will be close to *Q*. However, if most challengers are discriminators, a tough reputation, once earned by fighting, is “sticky”. Even a low-*Q*-defender can often keep a tough reputation for a long sequence of interactions since discriminators will continue to respect her property until a taker comes along and her reputation switches to weak again. This effect is not symmetric. A weak reputation is not sticky in this sense, since weak defenders’ resources keep being taken by discriminators.

The second reputation assessment scheme we consider is the *fighting-probability* scheme. Here, discriminators get rich information about the defender’s empirical past fighting frequency by observing a high number of past actions. In a limiting case this number is large enough to effectively inform the challenger of the defender’s fighting probability *Q*. An obvious way of classifying a defender as weak or tough is then for a discriminating challenger to use a threshold classification such as “*Classify defender as tough (and thus respect) if her empirical fighting frequency is higher than* $\bar{Q}$”, where $\bar{Q}$ is the threshold value. Hence, under this scheme, we have to distinguish between different discriminator strategies characterized by different $\bar{Q}$-values.

### Deterrence is never evolutionarily stable

A simple static analysis of reputation-based deterrence (see S1 Appendix) shows that, irrespective of the reputation assessment scheme, the only evolutionarily stable population state (ESS [28]) is the take-and-yield state without any deterrence. In such a state, building a reputation is doomed to fail. Mutant fighting defenders suffer from numerous costly fights, and mutant respecting challengers forego the large benefits of undisturbed taking. A state of successful deterrence where challengers discriminate and defenders are prepared to fight whenever their resources are taken (which, however, doesn’t happen), is not viable, since obtaining information on reputations is costly but useless for challengers facing a monomorphic defender population. The remaining possible deterrence scenario, where always-respecting challengers meet defenders ready to fight, is also unstable under selection. If defenders never have to actually react, their planned reactions are unconstrained by the forces of selection and can freely drift. Once enough yielders have accumulated, taking mutants can proliferate and deterrence breaks down. The static ESS analysis therefore fails to explain how reputation-based deterrence can survive in the long run.

This necessitates a closer look at the dynamics of the population game. We assume that individuals receive strategy revision opportunities at random points in time, and revising individuals best respond to the population state, but do so myopically, such that strategy revisions follow so-called best response dynamics [29–31]. This captures individuals’ bounded rationality better than the traditional replicator dynamics [32] that require genetic inheritance, reinforcement learning, or a special kind of imitative behavior [33–35]. Under best response dynamics, behavior in the two roles evolves independently, and the evolutionary dynamics can be analyzed as if challengers and defenders were members of two distinct populations.

### Deterrence may evolve with minimal information

**Fig 3:**
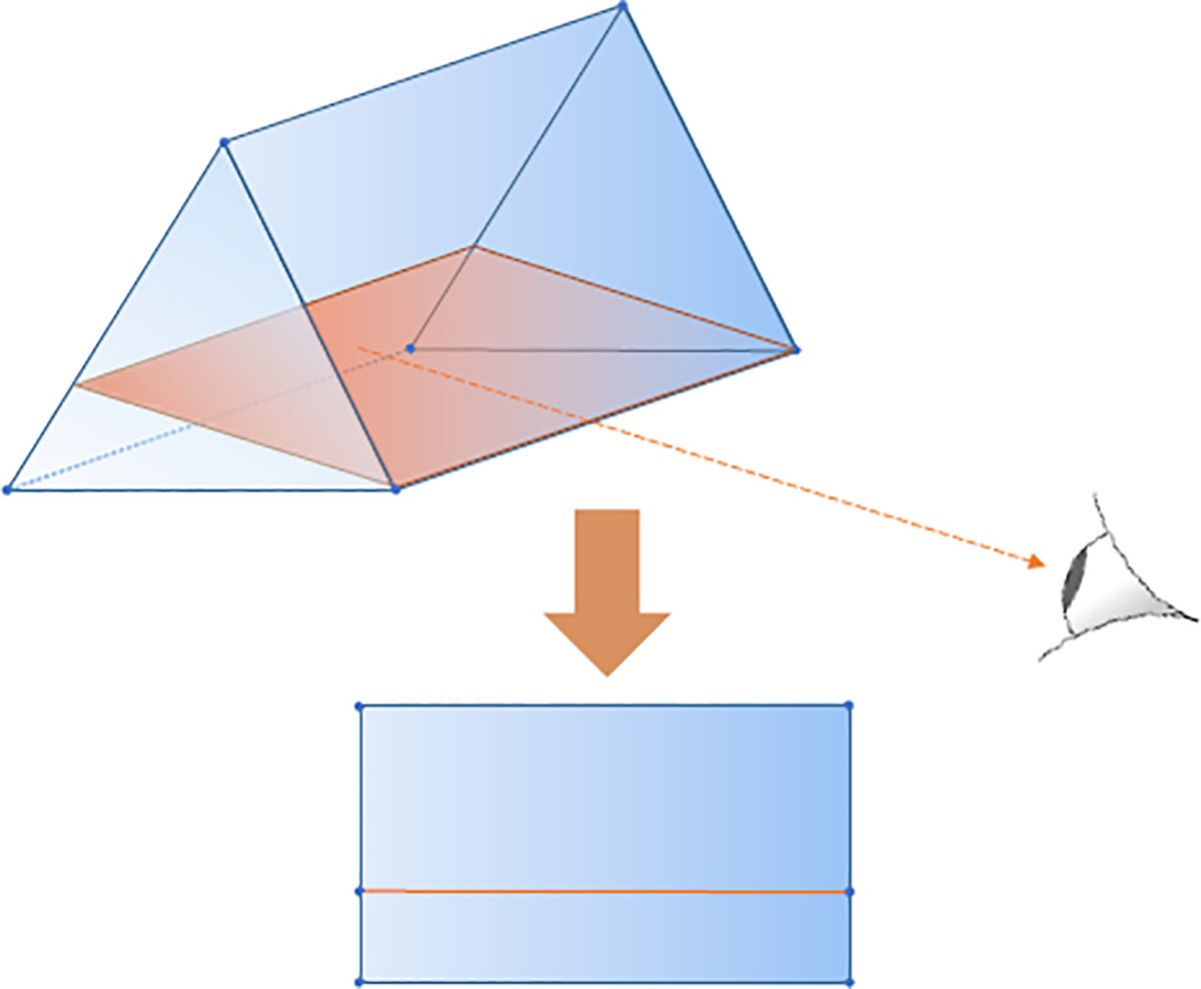
The two populations’ combined state space is a uniform triangular prism. This prism is divided into two regions by a plane on which fighters and yielders do equally well. Using the line of intersection of this plane with a triangular side of the prism as line-of-sight, the prism can be visualized as a rectangle in which the defenders’ indifference plane is a line parallel to the base of the rectangle.

**Fig 4:**
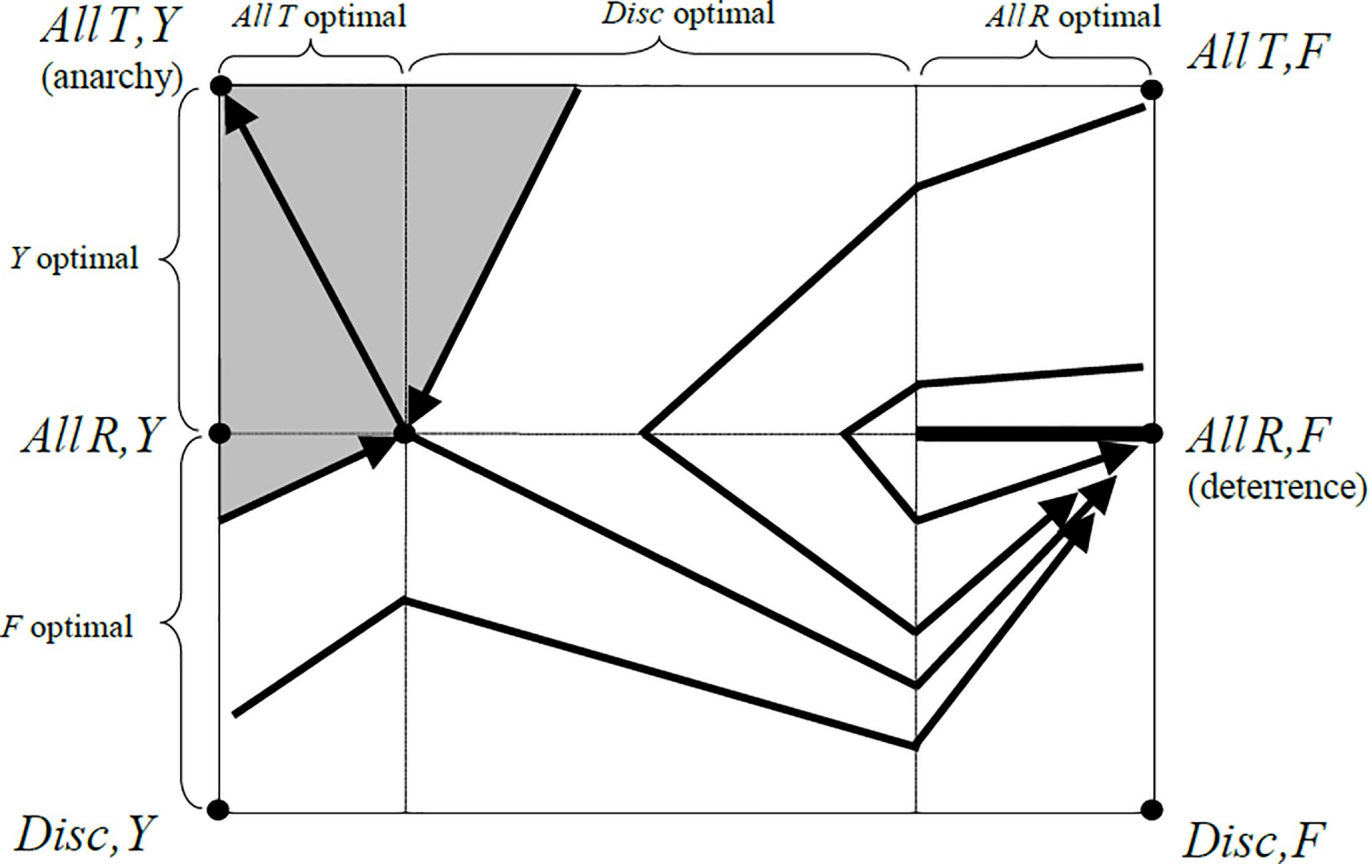
Projected best response paths for the evolutionary deterrence game. For initial population states close to the no-deterrence ESS (*AllT*,*Y*) deterrence fails to evolve. The shaded region is the basin of attraction of this anarchistic ESS. Outside this region, best response paths converge to the deterrence equilibrium at (*AllR*,*F*). This equilibrium is embedded in a stable component of equilibria with full deterrence. Discriminators die out in the long run, but they play an important role in steering evolving populations to full deterrence. Parameter values: *v* = *c* = 1, *a* = 0.2.

We first consider the last-action reputation assessment scheme (see S1 Appendix). We find that under this scheme, randomizing is always dominated by either fighting or yielding for defenders. After randomizers have disappeared, only pure fighters and pure yielders are left in the defender population. The state space can be projected to a rectangle to visualize the evolutionary dynamics in a convenient way, see Fig 3. This then allows the construction of the (projected) paths of the best response dynamics as explained in S1 Appendix and shown in Fig 4.

The state space is divided into two regions by the defenders’ indifference plane that appears as the interior horizontal line in Fig 4. Above the line, yielders do better than fighters and all paths move to the left. Below the line, the direction of best response paths is reversed. Unconditional taking is optimal if yielders are numerous, while unconditional respecting is optimal if most defenders are prepared to fight. For intermediate mixtures of yielders and fighters, challengers switch to discrimination. The shaded region in Fig 4 is the basin of attraction of the no-deterrence ESS (*AllT*,*Y*). This region is small for the (realistic) parameter range where the costs of information are small compared to the costs of fighting. Outside of the ESS’ attraction basin, populations converge to the (*AllR*,*F*) equilibrium. This equilibrium is part of a component of equilibria where challengers always respect and a large fraction of defenders are prepared to fight. While the polymorphic equilibria are individually unstable, the equilibrium component itself is evolutionarily stable. If a small fraction of individuals “mutates” and starts to experiment with different strategies, they are eventually driven back and the population returns to successful deterrence.

The game dynamics exhibit the decisive role of discrimination. While discriminators are completely absent in both attracting population states, discrimination is nevertheless responsible for the evolution of successful deterrence. Its presence, though only temporary, makes fighting worthwhile, and when discriminators finally disappear, fighters stay and challengers respect most of the time. In this sense discrimination acts as a catalyst for successful deterrence. As a consequence, reputation-based deterrence works well under quite minimal information, where observation of the last action only of a defender is possible at some cost. This corresponds to what can be observed in most real-world settings: We live under a norm of informal private property rights, where most of us do not even consider taking the belongings of others for personal gain, and even those who do consider it expect their opponents to fight for their possessions, and rightly so.

### Deterrence breaks down if information is too rich

Intuition suggests that if minimal information is good, rich information should be even better. Analysis of our second type of reputation assessment, the fighting-probability scheme, reveals that this intuition fails in the evolutionary deterrence game. The reason is that under the fighting-probability scheme, in contrast to the last-action scheme, randomizing may be optimal for defenders (see S1 Appendix). As long as randomizing defenders with diverse fighting probabilities are present, discriminators will tend to use Q* as their classification threshold. But once this threshold has become common among discriminators, defenders’ fighting probabilities cluster at Q* and eventually display so little diversity that discrimination loses its advantage and starts to decline. In the end, deterrence breaks down and the population converges to all-out take-and-yield. In this scenario, individuals are effectively victims of a social dilemma: While each individual challenger naturally prefers rich to minimal information, society-wide availability of rich information leads to anarchy.

### Agent-based simulations

The results presented here and proven in S1 Appendix have been derived from a simplified analytical model under strong assumptions and in limit scenarios: An infinitely large population, infinitely many interactions between strategy revisions, revising individuals exactly knowing the current population state and being perfectly able to calculate a best response, without noise and in the absence of errors in strategy execution or reputation assessment. It is thus questionable whether these results continue to hold under more realistic assumptions. Crucially, if defenders experiment with non-optimal strategies and if individuals have only a vague idea of which behaviors are optimal, their behavior becomes much more heterogeneous, which might invalidate some of the analytical arguments based on homogeneity of defenders. As a robustness check we therefore also conducted agent-based simulations which relax these strong assumptions and test for a variety of different parameter values.

Specifically, we modelled a large but finite population of *N* agents where initially all strategies, including the dominated ones, are present in roughly equal proportions. In each round, all agents are matched in pairs. Strategies for a given role may be switched if a revision opportunity arrives, which happens independently with probability *u* after each round for each agent. Information on reputations is subjected to noise by setting the defender’s image to the wrong one with some small probability *δ*. With another small probability, an agent commits an implementation error and plays the wrong action. Moreover, in each round, with some small probability *μ* an agent experiments and switches to a randomly chosen strategy. We keep track of each agent’s payoffs and after each round add the current payoff to the discounted sum of the agent’s past payoffs, using a discount factor *d*. This sum can be interpreted as an agent’s “wealth”, a stock variable which is easier to observe in a social group than payoff flows (“income”). Agents do not calculate best responses. Rather, each agent has a group of *N*_*F*_ friends whose wealth levels and strategies she monitors. Upon revising, an agent simply imitates the strategy with maximal average empirical wealth level among those used by her friends.

**Fig 5:**
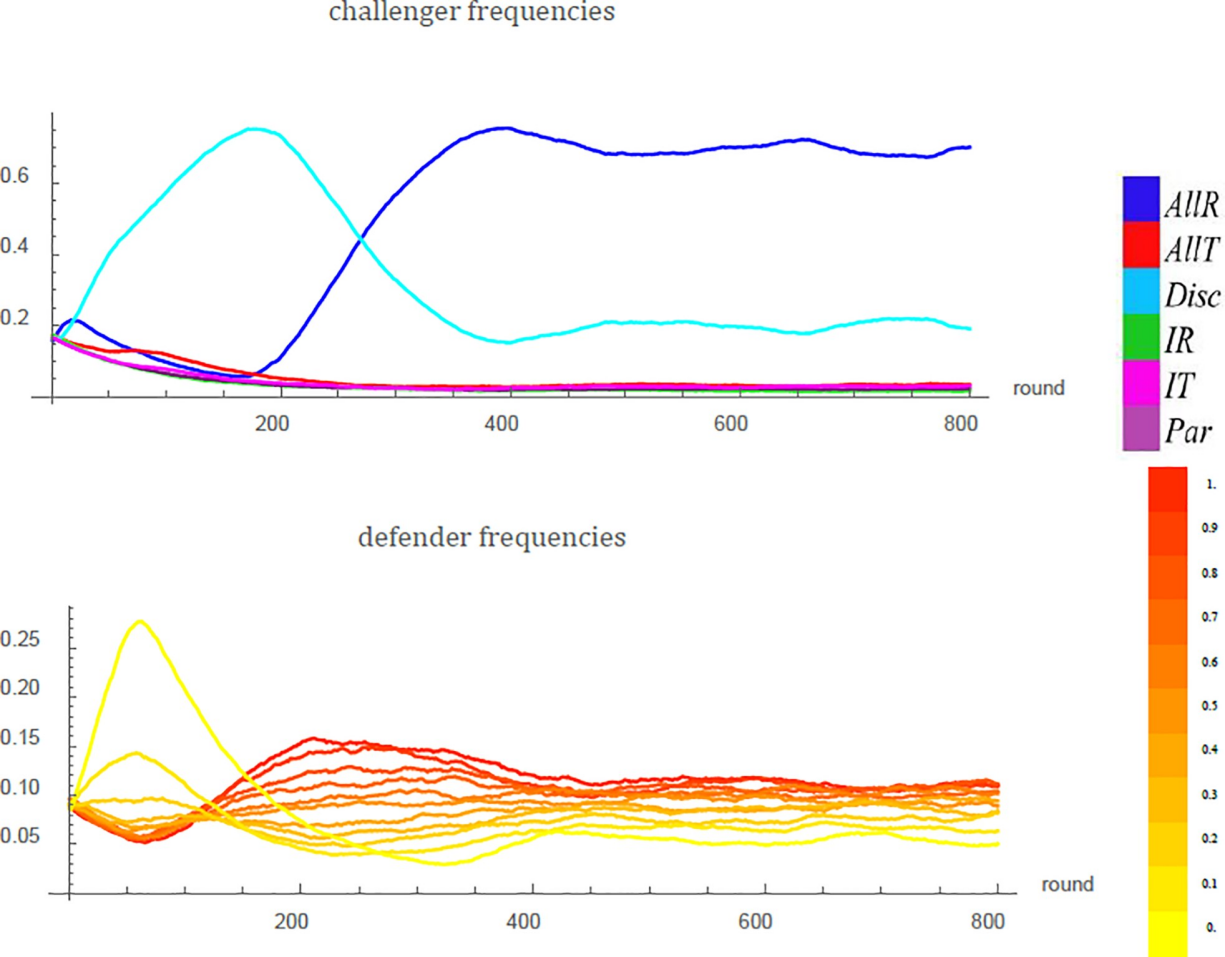
Evolution of deterrence in an example run of an agent-based simulation under the last-action reputation scheme. In the beginning, yielders (using strategy *Q* = 0, bright yellow) do best against the resident challengers, since takings are frequent. At the same time, discriminators (light blue) grow in frequency because discrimination pays off in the presence of a highly diverse defender population. However, this increases the value of having a tough reputation, and fighters (*Q* = 1, red) become more numerous. As soon as the defenders’ average fighting probability is high enough, unconditional respecting (dark blue) takes over as it avoids paying the information costs. The frequency of this strategy oscillates around approximately 0.7, followed by discrimination with a frequency of approximately 0.2. Defenders’ property is mostly respected and just a small amount of taking and fighting remains. Parameter values: *v* = 1, *c* = 1.5, *a* = 0.1, *N* = 10^4^, *N*_*F*_ = 100, *d* = 0.9, *u* = 10^−2^, *μ* = *ϵ* = *δ* = 10^−3^.

**Fig 6:**
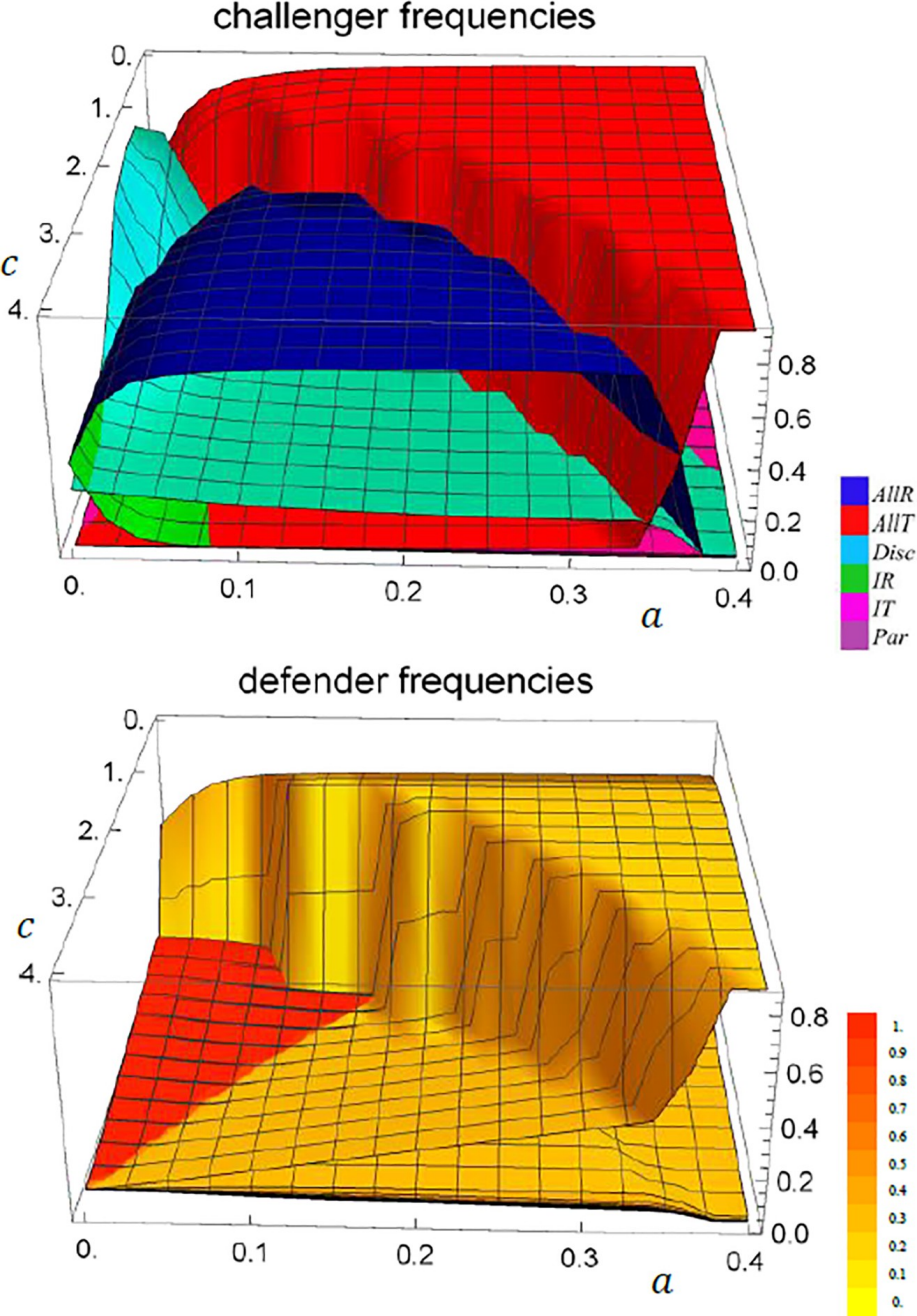
Long term challengers’ and defenders’ strategy frequencies if reputations are determined by last-action. If the costs *c* of fighting are low, deterrence fails and takers and yielders prevail. For high fighting costs, most challengers respect and deterrence is able to evolve. For intermediate fighting costs deterrence can also be based on discrimination if information costs are very small. Successful deterrence leads to low selection pressure for defender strategies and fighting probabilities vary widely. Parameter values: *v* = 1, *N* = 10^4^, *N*_*F*_ = 100, *d* = 0.9, *u* = 10^−2^, *μ* = *ϵ* = *δ* = 10^−3^. Frequencies are averaged over rounds 8000−10000 from 100 runs.

The results we obtained from agent-based simulations corroborate the implications of our analytical model. Under the last-action reputation scheme, single runs eventually either hover around a state where most takings are successfully deterred (Fig 5) or converge to the vicinity of the no-deterrence ESS, depending on parameters. If information costs *a* are small relative to fighting costs *c*, deterrence prevails in the long run (Fig 6).

Increasing an agent’s “memory length” allows us to interpolate between our two extreme reputation assessment schemes. Let us assume that for any given memory length *k* discriminators always use the classification scheme according to whether or not a defender’s empirical fighting frequency among her last *k* reactions exceeds the critical value Q*.

**Fig 7:**
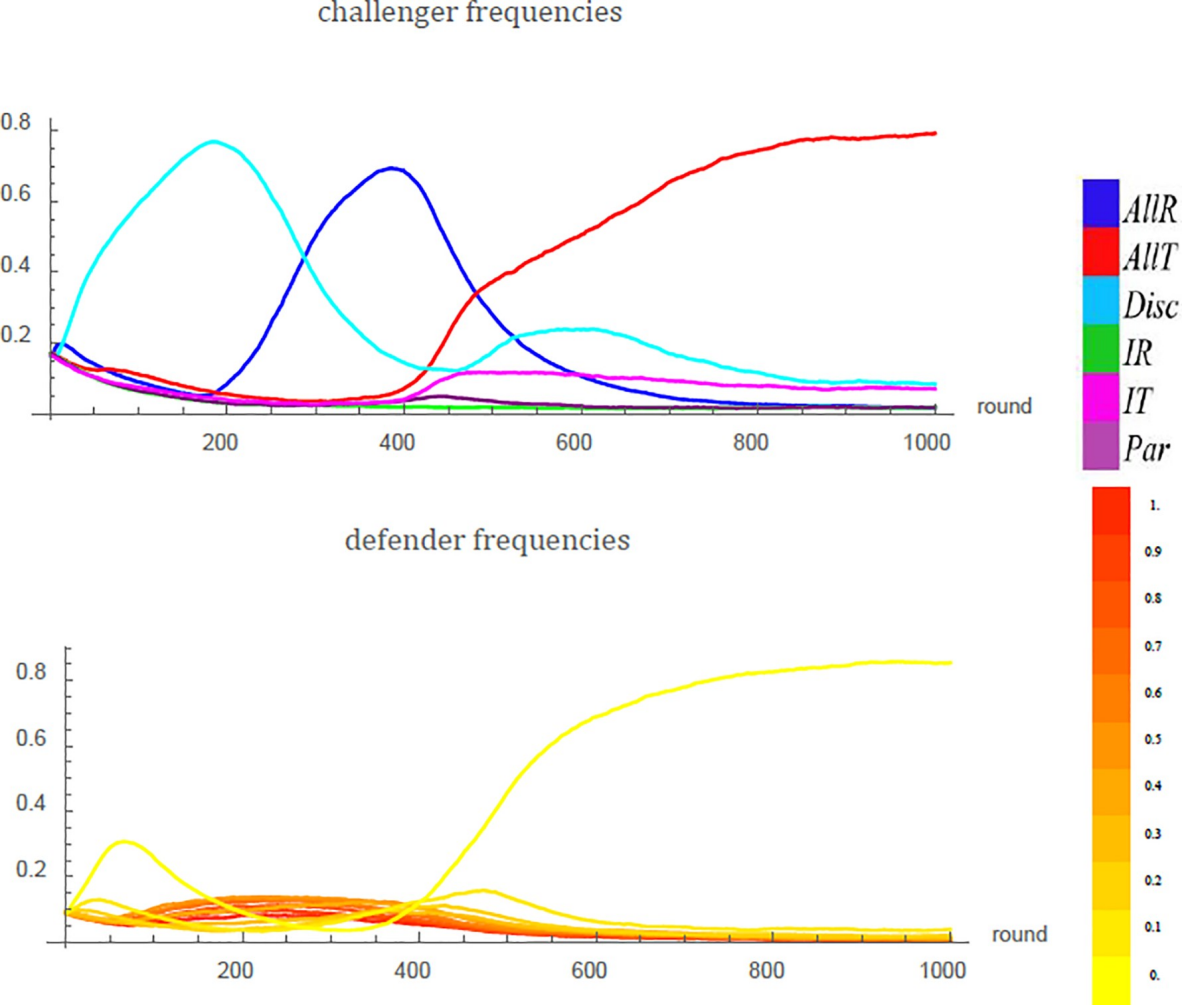
The breakdown of deterrence in an example run under the (empirical) fighting-frequency scheme proceeds over three stages. (i) In the beginning, yielders (strategy *Q* = 0, bright yellow) do best against the resident challengers, since taking is frequent. At the same time, discriminators (light blue) grow in frequency because discrimination pays off in the presence of a highly diverse defender population. (ii) When discriminators are numerous, yielding gives way to intermediate fighting probabilities close to Q* (orange) for defenders. Discrimination loses its value and is displaced by respecting (dark blue), thereby (iii) initiating a comeback of yielders. This invites unconditional takers (red) who eventually take over the challenger role, while yielding dominates the defender role. Parameter values: *v* = 1, *c* = 1.5, *a* = 0.1, *N* = 10^4^, *N*_*F*_ = 100, *d* = 0.9, *u* = 10^−2^, *μ* = *ϵ* = *δ* = 10^−3^. Here, Q* = 0.4 and 35 past actions are observed.

Then *k* = 1 corresponds to the last-action scheme while large values of *k* approximate the fighting-probability scheme, provided strategy revisions are sufficiently rare. With a higher number of past actions observed, discriminators get richer information and therefore more precise estimates of their matched defenders’ fighting probability *Q*, but the ensuing tendency of these fighting probabilities to flock close to Q* weakens challengers’ incentives to invest in discrimination and therefore decreases the prospects of deterrence (Fig 7).

**Fig 8:**
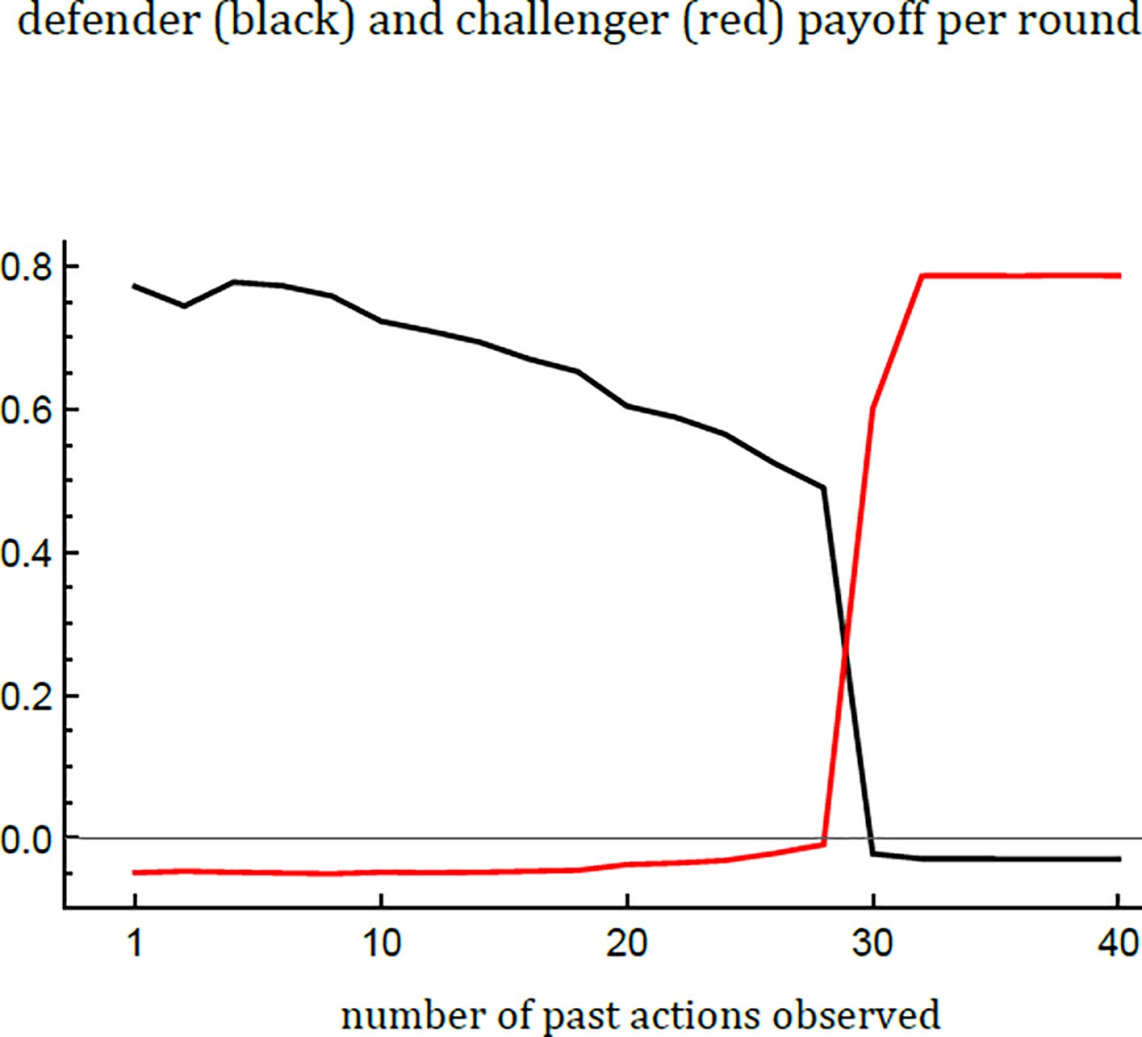
Dependence of average challenger and defender long term payoffs on the number of observations if reputations are determined by empirical fighting frequencies. With only a single observation, the fighting-frequency scheme coincides with the last-action scheme. Defender payoff is high if the number of observations is small and deterrence can evolve. As the number of observations grows, defender payoff slowly declines first and then suddenly drops below zero when deterrence breaks down. For challengers, the situation is reversed. Parameter values: *v* = 1, *c* = 1.5, *a* = 0.1, *N* = 10^4^, *N*_*F*_ = 100, *d* = 0.9, *u* = 10^−2^, *μ* = *ϵ* = *δ* = 10^−3^. Payoffs are averaged over rounds 8000−10000 from 100 runs.

Our simulations show that defenders’ average payoffs decline approximately linearly in memory length until at some critical number of past actions observed, deterrence suddenly breaks down completely (Fig 8).

## Discussion

The proposed mechanism behind the evolution of deterrence based on reputation is reminiscent of the explanation of the evolution of cooperation by indirect reciprocity. These models are based on the prisoner’s dilemma or its one-sided variant, the donation game, where a donor can pay a small cost to provide a large benefit to a passive recipient. Discriminators withhold cooperation (donating) with individuals who have been observed to defect (denying donations) in the past, thereby providing others with an incentive to cooperate [26, 36–39].

Withholding donations to previous defectors might be viewed as thirdparty retaliation, but this mechanism suffers from the problem of justified defection: If a discriminator defects against a defector, she might earn a bad reputation herself. No such difficulties arise in the deterrence game. Individuals in the role of a challenger discriminate based on their opponent’s past behavior as defenders only, and the separation of these two roles avoids the need to distinguish between “justified” and “unjustified” retaliations.

### Evolution of costly punishment and fairness

Costly punishment [11, 40] has mainly been studied as an add-on to a prisoner’s dilemma or a public goods game [23, 41–45]. It has been shown, however, that stabilizing cooperative social outcomes by threats of punishment often fails to work, and costly punishment has therefore been suggested to have evolved for other reasons [46, 47]. In our model, retaliation can be viewed as a special variant of costly punishment that is not confounded with decisions of whether or not to cooperate in a prisoner’s dilemma or a public goods game.

Our study suggests that an explanation for costly punishment might be found in its value as a reputation-based deterrence device in environments where recent punishment events are observable at some cost. As our results also apply to the Mini-Ultimatum Game variant of deterrence, an interpretation of reputation-based deterrence as a possible mechanism for the evolution of “fairness” is suggestive. By unswervingly rejecting unfair offers responders acquire a reputation as tough bargainers, allowing fair offers to prevail in the long run.

### Evolution of private property rights

Legal scholars are well aware of the hypothesis that modern property law has roots in evolutionary biology [48, 49], and biologists have studied animal territoriality for decades: Individuals of many species seem to grant ownership of a territory to whichever individual arrived there first. This phenomenon has been famously explained by the *bourgeois strategy* which is an ESS of the Hawk-Dove-Bourgeois game and prescribes to play Hawk if owner and Dove if intruder [50].

However, there is a fundamental symmetry in the Hawk-Dove game, by which there exists another strategy, the anti-bourgeois strategy, prescribing the exact opposite behavior, Dove if owner, and Hawk if intruder [28, 51]). This strategy also constitutes an ESS that has been called the paradoxical ESS or the anti-private-property equilibrium, since the associated behavior is counterintuitive and almost nonexistent in nature. The fundamental symmetry of the Hawk-Dove game does not allow for a satisfactory explanation for the absence of these anti-private-property equilibria unless one introduces exogenous asymmetries between owners and intruders into the model, such as differing valuations of the property, differing contestability, or an endowment effect [52, 53].

Theorists trying to explain the prevalence of the private-property equilibrium have developed sophisticated game-theoretic models focusing on the behavior of nonhuman animals competing for territories. These models are mainly built on variants of the Hawk-Dove game [54–57]. Our model aims to describe human behavior and therefore relies on a fundamentally different mechanism, reputation-based deterrence, to explain the evolution of informal property rights. While we cannot eliminate the no-deterrence ESS that corresponds to the anti-private-property equilibrium, our dynamic analysis reveals that its basin of attraction is quite small for small information costs. This might be a reason why the anarchistic ESS is unlikely to get established in human populations.

## Supporting information

S1 Appendix Analytical results for the general payoff structure. (PDF)
