# Supplementary material for: Evolution of deterrence with costly reputation information
Source: PLoS One. 2021 Jun 15;16(6):e0253344. doi: 10.1371/journal.pone.0253344 (PMC8205128; doi:10.1371/journal.pone.0253344)
Supplement: S1 Appendix — (PDF) [file pone.0253344.s001.pdf]

S1 Appendix: Analytical Results for the General Payoff Structure

Analytical results for the last-action reputation assessment scheme

For the general payoff structure of the deterrence game we use parameters v and c for defenders and v' and c' for challengers as shown in Fig. A1. The latter are then indifferent between taking and respecting if the defender's fighting probability is $Q^* = \frac{v'}{c'+v'}$. We first consider only pure strategies. Denote the challengers' pure strategy set by $S_1 = \{AllR, AllT, Disc, IR, IT, Par\}$, and let $S_2 = \{F, Y\}$ be the defenders' pure strategy set. The payoff for an individual playing $(B, C) \in S = S_1 \times S_2$ against an individual playing $(D, E) \in S$ is given by $\pi\big((B, C), (D, E)\big) = \frac{1}{2}\pi_1(B, E) + \frac{1}{2}\pi_2(D, C)$, where the payoff functions π_1 and π_2 are the ones of challenger and defender, respectively, in the asymmetric evolutionary deterrence game, given by the payoff bimatrix $(\pi_1, \pi_2) =$

	F	Y
$AllR$	$0, v$	$0, v$
$AllT$	$-c', -c$	$v', 0$
$Disc$	$-a, v$	$v' - a, 0$
IR	$-a, v$	$-a, v$
IT	$-c' - a, -c$	$v' - a, 0$
Par	$-c' - a, -c$	$-a, v$

From the payoff bimatrix it is clear that the last three challenger strategies are strictly dominated. We therefore henceforth ignore these strategies here. Inspection reveals that

the pure-strategy Nash equilibria are (*AllR, F*) and (*AllT, Y*). Only the latter one is strict, however. Since in asymmetric games (and their symmetrizations), ESS' correspond to strict Nash equilibria, there is only a single ESS, the strategy pair $(AllT, Y)$, in which deterrence fails.

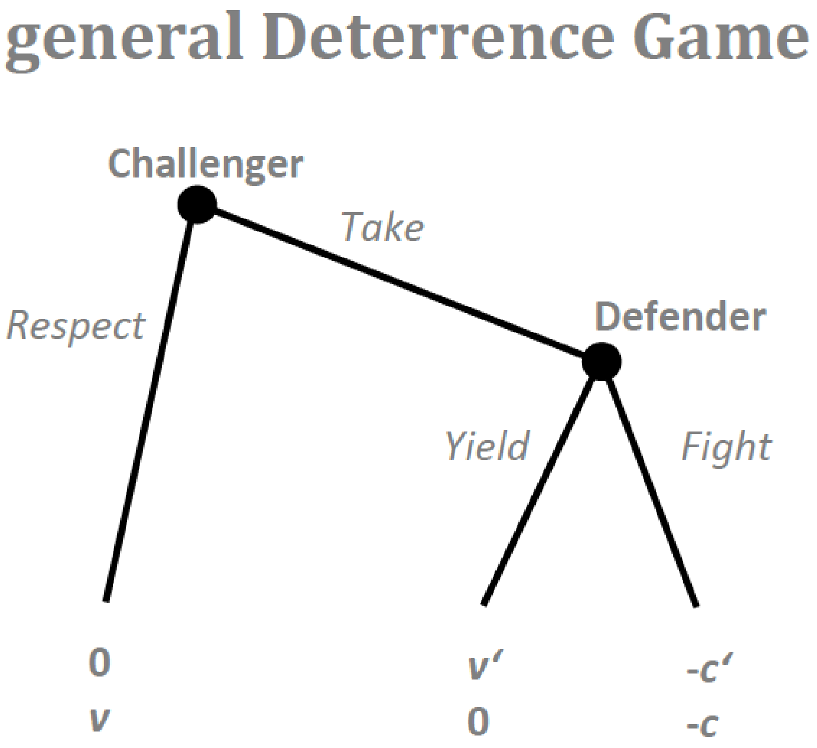

Fig. A1. The general payoff structure of the deterrence game. The value of the resource and the costs of fighting, respectively, may be different for challengers and defenders.

Let $\Delta(S_1)$ be the space of probability distributions p on S_1. Now imagine a Q-defender playing against a challenger population in state $p \in \Delta(S_1)$. Let g be her current probability of having a tough reputation. Then after her next match her probability g' of having a tough reputation can be calculated by considering the following: Having a tough reputation after the match can result from three different scenarios. First, the defender initially had a tough reputation and was matched with an *AllR*- or a *Disc*-challenger, thus being respected, which leaves her reputation unchanged. This occurs with probability

$g(p_{AllR} + p_{Disc})$. Second, she initially had a tough reputation, got matched with an *AllT*-challenger and reacted by fighting. This has probability $gp_{AllT}Q$. Third, she initially had a weak reputation but met an *AllT*- or a *Disc*-challenger and reacted by fighting. The probability for this chain of events is $(1-g)(p_{AllT} + p_{Disc})Q$. Adding these three probabilities results in $g' = (p_{AllR} + p_{Disc} - Qp_{Disc})g + Q(p_{AllT} + p_{Disc})$. Iterating this procedure for constant p shows that for a Q-defender the probability of having a tough reputation converges to a fixed point given by$g(p, Q) = \frac{Q(p_{AllT}+p_{Disc})}{p_{AllT}+Qp_{Disc}}$, whenever the denominator is nonzero. For simplicity we assume from now on that $p_{AllT} > 0$, which ensures that $g(p, Q)$ is always well-defined. $g(p, Q)$ increases monotonically in Q from $g(p, 0) = 0$ to $g(p, 1) = 1$.

The expected payoff for a Q-defender depends on $g(p, Q)$ and therefore is nonlinear in Q. A Q-defender receives v if she meets an *AllR*-challenger or if she meets a discriminator and has a tough reputation, thus being respected. She loses c whenever she either meets a taker and fights back or she meets a discriminator when having a weak reputation and fights back. Combining the corresponding probabilities results in a payoff for a Q-defender of $\pi_2(p, Q) = [p_{AllR} + g(p, Q)p_{Disc}]v - [p_{AllT} + p_{Disc} - g(p, Q)p_{Disc}]cQ$.

Best response dynamics

We assume that strategy updating is guided by the social learning process known as the best-response dynamics (BR-dynamics) [29–31]. Since the BR-dynamics is defined for games with finitely many pure strategies, we define $S_Q = \{0, Q^*, 1, \dots\}$ to be any finite discretization of the defenders' pure strategy space including at least 0, 1, and Q^*. Let $\Delta(S_Q)$ be the space of mixed strategies, i.e. probability distributions over SQ. Denoting the population state by $x(t) \in \Delta(S_1 \times S_Q)$, this results in $x(t)$ moving along (possibly non-

unique) solutions, called best response paths (BR-paths), of the differential inclusion $\dot{x}(t) \in BR(x(t)) - x(t)$, where $BR(x)$ is the set of (pure or mixed) best responses to the population state x. As long as the current best response is unique, the BR-path describes a straight line in the state space pointing to the current pure best response. If a BR-path converges, the limit is a Nash equilibrium.

We assume two separate time-scales here: a slow one for the best response dynamics of the population state x and a fast one for the adaptation of the reputations of Q-defenders to their stationary values for fixed x. This allows us to assume that reputations are instantly equilibrated while the population state moves through the state space.

Now we set out to find the long-run behavior of the population state in the symmetrized game under BR-dynamics. Though the state space $\Delta(S_1 \times S_Q)$ has dimension $3|S_Q| - 1$ (which is at least 8), the task is greatly simplified by the following two steps, which we use to sequentially reduce the state space we have to analyze.

Step 1: De-symmetrizing the game

Consider the projection from $\Delta(S_1 \times S_Q)$ to $\Delta(S_1) \times \Delta(S_Q)$ which separates a mixed strategy $x \in \Delta(S_1 \times S_Q)$ into its corresponding pair of marginal distributions $(p(x), q(x)) \in \Delta(S_1) \times \Delta(S_Q)$. This projection respects the best response structure of the game and therefore maps BR-paths $x(t)$of the symmetrized game to BR-paths $(p(t), q(t))$ of the corresponding asymmetric game given by the role-contingent payoff functions π_1 and π_2, as demonstrated in [1]. With a little abuse of notation, we stick to denoting the defender's payoff function by π_2 when we extend her strategy space from S_2 to S_Q by including all feasible mixed strategies Q. Here, $(p(t), q(t))$ is a BR-path of the corresponding asymmetric (two-populations) game, i.e. a solution to the system of

differential inclusions $(\dot{p}(t),\dot{q}(t)) \in (B_1(q(t)), B_2(p(t))) - (p(t), q(t))$, where B_1 and B_2 are the respective best response correspondences of challengers and defenders in the asymmetric evolutionary deterrence game.

From the long-run behavior of the population state in this game we can then infer the long-run behavior of the population state in the symmetrized game. This allows us to reduce the dimension of the state space we are working in from $3|S_Q| - 1$ to $2|S_Q| - 2$.

Step 2: Eliminating strictly dominated strategies

Under BR-dynamics updating players never switch to strictly dominated strategies, hence such strategies are eliminated quickly. It thus suffices to study the BR-dynamics in the reduced evolutionary deterrence game after eliminating strictly dominated strategies. Note that we have already discarded the challengers' strictly dominated strategies *IR*, *IT*, and *Par*. Turning to defenders, substituting for $g(p,Q)$ and for p_{AllR} in the expression for a Q-defender's expected payoff derived above, differentiating w.r.t. Q and simplifying yields $\frac{\partial \pi_2}{\partial Q}(p,Q) = \frac{p_{AllT}(p_{AllT}+p_{Disc})v}{(p_{AllT}+Qp_{Disc})^2}(p_{Disc} - \frac{c}{v}p_{AllT})$.

It follows that there exists an indifference hyperplane $M := M_1 \times \Delta(S_Q)$, with $M_1 := \{p \mid p_{Disc} = \frac{c}{v}p_{AllT}\}$, that separates the state space into two regions. Therefore, if $p_{Disc} > \frac{c}{v}p_{AllT}$, then the defender's payoff is strictly increasing in Q, while for $p_{Disc} < \frac{c}{v}p_{AllT}$ it is strictly decreasing. In the indifference hyperplane, the defender's payoff is given by $\pi_2(p,Q) = v - p_{AllT}(c+v)$ for all Q. As a consequence, whenever the population state is not in the hyperplane M, a defender's best response is either $Q = 0$ or $Q = 1$, i.e. Y or F, while all $Q \in S_Q$ are optimal if $(p,q) \in M$.

Allowing defenders to randomize thus turns out to be inconsequential for the model, since randomizing, i.e. using $0 < Q < 1$, is only optimal if playing a pure strategy F or Y is also optimal. (The same result holds trivially for randomizing challengers, since their expected payoffs are linear functions of their randomization probabilities anyways.) We can therefore proceed under the simplifying assumption that individuals use only pure strategies from the outset. Eliminating the unused randomizing strategies from the game results in the reduced evolutionary deterrence game. We again simplify notation by sticking to π_1 and π_2 for the payoff functions and return to denoting the pure strategy sets by S_1 and S_2. The two-population version of this game is a standard evolutionary bimatrix game comprising the strategies *AllR*, *AllT*, and *Disc* for challengers and F and Y for defenders. It is given by the payoff bimatrix $(\pi_1, \pi_2) =$

	F	Y
AllR	$0, v$	$0, v$
AllT	$-c', -c$	$v', 0$
Disc	$-a, v$	$v' - a, 0$

The reduced game is only interesting if obtaining information is not prohibitively costly, i.e. if *Disc* is not dominated by a mixture of *AllR* and *AllT*. A quick calculation shows that for this we have to assume $a < \frac{v'c'}{v'+c'}$, which we will do henceforth. We have now reduced the dimension of the state space from at least 8 to 3. The remaining task is to solve for the long-run behavior of the BR-dynamics.

The reduced evolutionary deterrence game is a two-person game where one of the players has only two strategies. For this class of games it is known that all BR-paths converge to the set of Nash equilibria. Moreover, a suitable projection $P: \Delta(S_1) \times \Delta(S_2) \rightarrow$

$[0,1] \times [-v, c]$ allows one to analyze the global dynamics in these games in two dimensions (see [2] for details). The projection P is chosen in such a way that the plane of indifference of the defender is projected to the horizontal axis, corresponding to the visualization in the main text. With a slight abuse of notation, we denote this plane by M again. P maps the state space $\Delta(S_1) \times \Delta(S_2)$ and its partition into 6 best response regions to the rectangle $[0,1] \times [-v, c]$, partitioned into 6 rectangles in the plane. For population states $(p, q) = \big((p_{AllR}, p_{AllT}, p_{Disc}), (q_F, q_Y)\big) \in \Delta(S_1) \times \Delta(S_2)$ the projection map P is given by $P(p, q) = (q_F, \delta(p))$, where $\delta(p) := \pi_2(p, Y) - \pi_2(p, F)$ is the defenders' payoff advantage of Y over F if the challengers' population state is p. We call $P(p, q)$ the *induced population state*.

P is linear and therefore maps BR-paths $(p(t), q(t))$ in the reduced game to piecewise linear so-called induced paths $(q_F(t), \delta(p(t)))$ in the rectangle $[0,1] \times [-v, c]$. The behavior of these induced paths is easy to study and allows one to obtain the behavior of BR-paths in the reduced game and, by reversing Steps 2 and 1 of the reduction of dimensions above, in the asymmetric evolutionary deterrence game and finally in the original symmetric evolutionary deterrence game.

Since the projection map P is linear, piecewise linear BR-paths pointing to pure strategy pairs are mapped to piecewise linear induced paths pointing to points on the boundary of the induced state space (the rectangle $[0,1] \times [-v, c]$) in the plane. The remaining analysis of induced paths amounts to a simple exercise in planar geometry as shown in Fig. 4 in the main text.

By construction of P, defenders switch to yielding if the induced population state is above the horizontal axis and they switch to fighting if it is below the horizontal axis. Induced paths therefore move to the left above and to the right below the horizontal axis.

If q_F is large, i.e. if most defenders are prepared to fight, challengers switch to always respecting (*AllR*), since neither taking nor obtaining information pays off. In the rightmost vertical sector, therefore, induced paths point to one of the boundary points of the horizontal axis. This is the case for $q_F > 1 - \frac{a}{v'}$. If q_F is in the intermediary range $\frac{a}{c'} < q_F < 1 - \frac{a}{v'}$, the discriminating strategy *Disc* becomes optimal for challengers. In the middle vertical sector induced paths therefore point to one of the bottom vertices of the rectangle. Finally, if q_F is small enough, $q_F < \frac{a}{c'}$, it does no longer pay to discriminate and challengers turn to always taking (*AllT*). In the leftmost vertical sector induced paths therefore point to one of the top vertices of the rectangle.

The payoff bimatrix of the reduced game shows that it admits a strict Nash equilibrium at $(AllT, Y)$. This Nash equilibrium is asymptotically stable under BR-dynamics, since it is the unique best response to all nearby population states. It constitutes the no-deterrence ESS where challengers always take and defenders always yield. However, a quick calculation shows that for small information costs a the relative size of this equilibrium's basin of attraction, depicted as the shaded region in Fig 4, is about $a\frac{c'+2v'}{2v'(c'+v')}$, which shrinks to zero with a. The mixed equilibrium with $q_F = \frac{a}{c'}$, $p_{AllR} = 0$, $p_{AllT} = \frac{v}{c+v}$, and $p_{Disc} = \frac{c}{c+v}$, is saddle-like and therefore unstable. Its stable manifold separates the basins of attraction of the anarchistic ESS and the deterrence equilibrium.

The deterrence equilibrium $(AllR, F)$ is only semistable: In a small neighborhood it attracts all paths from the half-space with $\delta(p) < 0$ and repels all paths with $\delta(p) > 0$. But this equilibrium is part of a 1-dimensional equilibrium component $\left\{(AllR, q) |\ q_Y \leq \frac{a}{v'}\right\}$, which itself is asymptotically stable and has a large basin of attraction for small a. The equilibria in this component are behaviorally indistinguishable, since defenders' property rights are always respected. The equilibrium $(AllR, F)$ attracts all paths in some

neighborhood of this component, excluding the component itself. Within the equilibrium component, movement is indeterminate as there are infinitely many BR-paths originating from each equilibrium, moving back and forth arbitrarily within the component.

The analysis also shows that if we let $a \to 0$, there is a discontinuity in the long-run outcome. While for arbitrarily small $a > 0$ the population always typically ends up at $(AllR, F)$, this is not the case for completely costless information: If $a = 0$, *Disc* becomes a weakly dominant strategy for challengers and all interior BR-paths converge to $(Disc, F)$.

Analytical results for the fighting-probability reputation assessment scheme

As shown above, for a defender randomizing with fighting probability Q the probability of her last action having been F is given by $g(p, Q) = \frac{Q(p_{AllT}+p_{Disc})}{p_{AllT}+Qp_{Disc}}$. For all other past actions, the corresponding probability is simply Q. Thus, her empirical fighting frequency calculated from the last k actions converges to Q as $k \to \infty$, provided that, as we assume here, her last switch appeared before those k actions were carried out. In the limiting case, a discriminator is therefore informed of the defender's true fighting probability Q. The optimal strategy for a discriminating challenger is then to take if $Q < Q^*$ and to respect if $Q > Q^*$, i.e. to use the Q^*-threshold strategy. We denote this strategy by *Disc* again. Against a taker, yielding $(Q = 0)$ is the best response for defenders and against a *Disc*-challenger it is clearly optimal to use Q^* (assuming w.l.o.g. that discriminators respect if indifferent). All other fighting probabilities are never optimal except if there are no takers in the challenger population.

After eliminating the suboptimal fighting probabilities for defenders, the payoff bimatrix of the remaining strategies is given by $(\pi_1, \pi_2) =$

	Q^*	Y
AllR	$0, v$	$0, v$
AllT	$v' - Q^*(v' + c'), -cQ^*$	$v', 0$
Disc	$-a, v$	$v' - a, 0$

Substituting for Q^* this payoff bimatrix becomes $(\pi_1, \pi_2) =$

	Q^*	Y
AllR	$0, v$	$0, v$
AllT	$0, -cv'/(v' + \mathrm{c}')$	$v', 0$
Disc	$-a, v$	$v' - a, 0$

It follows that always taking is weakly dominant for challengers. Therefore, all interior BR-paths converge to the anarchistic ESS $(AllT, Y)$.

References

1. Berger, U. Best response dynamics for role games. *International Journal of Game Theory* **30,** 527–538 (2002).
2. Berger, U. Fictitious play in 2×n games. *Journal of Economic Theory* **120,** 139–154 (2005).
